# Uncompensated care provided by for-profit, not-for-profit, and government owned hospitals

**DOI:** 10.1186/1472-6963-10-90

**Published:** 2010-04-07

**Authors:** Peter Cram, Levent Bayman, Ioana Popescu, Mary S Vaughan-Sarrazin, Xueya Cai, Gary E Rosenthal

**Affiliations:** 1 Division of General Internal Medicine, Department of Internal Medicine, University of Iowa Carver College of Medicine, Iowa City, IA, USA; 2 Center for Research in the Implementation of Innovative Strategies for Practice (CRIISP), Iowa City Veterans Administration Medical Center, Iowa City, IA, USA

## Abstract

**Background:**

There is growing concern certain not-for-profit hospitals are not providing enough uncompensated care to justify their tax exempt status. Our objective was to compare the amount of uncompensated care provided by not-for-profit (NFP), for-profit (FP) and government owned hospitals.

**Methods:**

We used 2005 state inpatient data (SID) for 10 states to identify patients hospitalized for three common conditions: acute myocardial infarction (AMI), coronary artery bypass grafting (CABG), or childbirth. Uncompensated care was measured as the proportion of each hospital's total admissions for each condition that were classified as being uninsured. Hospitals were categorized as NFP, FP, or government owned based upon data obtained from the American Hospital Association. We used bivariate methods to compare the proportion of uninsured patients admitted to NFP, FP and government hospitals for each diagnosis. We then used generalized linear mixed models to compare the percentage of uninsured in each category of hospital after adjusting for the socioeconomic status of the markets each hospital served.

**Results:**

Our cohort consisted of 188,117 patients (1,054 hospitals) hospitalized for AMI, 82,261 patients (245 hospitals) for CABG, and 1,091,220 patients for childbirth (793 hospitals). The percentage of admissions classified as uninsured was lower in NFP hospitals than in FP or government hospitals for AMI (4.6% NFP; 6.0% FP; 9.5% government; P < .001), CABG (2.6% NFP; 3.3% FP; 7.0% government; P < .001), and childbirth (3.1% NFP; 4.2% FP; 11.8% government; P < .001). In adjusted analyses, the mean percentage of AMI patients classified as uninsured was similar in NFP and FP hospitals (4.4% vs. 4.3%; P = 0.71), and higher for government hospitals (6.0%; P < .001 for NFP vs. government). Likewise, results demonstrated similar proportions of uninsured patients in NFP and FP hospitals and higher levels of uninsured in government hospitals for both CABG and childbirth.

**Conclusions:**

For the three conditions studied NFP and FP hospitals appear to provide a similar amount of uncompensated care while government hospitals provide significantly more. Concerns about the amount of uncompensated care provided by NFP hospitals appear warranted.

## Background

For decades policy makers, researchers, and regulators have debated the advantages and disadvantages of for-profit (FP), not-for-profit (NFP), and government owned hospitals [1-5] Critics of FP hospitals have argued that such hospitals, beholden to investors, deliver care that is more expensive [6-8] and lower quality than hospitals with other models of ownership [9-11]. Supporters of FP hospitals have countered that a lack of financial accountability allows NFP and government hospitals to be less efficient than FP hospitals and that available data do not support allegations that FP hospitals consistently deliver lower quality care [12-17].

The debate over hospital ownership has been revitalized because of recent allegations that many NFP hospitals are not providing adequate amounts of uncompensated services (a.k.a., community benefit) to justify the exemptions from federal and state taxes that they receive [18-21]. Recent reports in the lay-press have chronicled aggressive profit-seeking behaviors by certain NFP hospitals that seem inconsistent with federal and state tax subsidies that these hospitals receive. NFP hospitals have been faulted for selectively closing facilities located in poor urban neighborhoods and spending lavishly on capital improvement and generous executive salaries [22,23]. Policymakers have responded to these allegations with government investigations and proposals for regulatory reform. Most notably, a number of current healthcare reform proposals include legislation that would mandate that NFP hospitals provide an explicitly defined quantity of charity care (e.g., 5% of total revenue) or risk losing their tax exempt status [24,25]. Despite these concerns, there are relatively limited data comparing the quantity of uncompensated care provided by NFP, FP and government owned hospitals [26,27]. Moreover, most of the available literature comparing the quantity of uncompensated care provided by hospitals with differing ownership structure have relied upon aggregated hospital-level financial reports rather than analysis of actual patient-level insurance coverage; there are legitimate concerns about the fidelity of hospital-level financial reporting of uncompensated care given the strong existing regulatory pressures on NFP hospitals not to report excess profitability and/or exceedingly low levels of uncompensated care or risk attracting the attention of state and federal regulators [28].

Our objective was to examine the amount of uncompensated care provided by FP, NFP, and government owned hospitals by evaluating the insurance coverage of patients hospitalized with three common medical diagnoses (acute myocardial infarction [AMI], coronary artery bypass grafting [CABG], and childbirth). These diagnoses were selected because the conditions represented a spectrum ranging from higher acuity (AMI and childbirth) to lower acuity (CABG) and affect different patient populations. Thus we expected that our study of these three diagnoses would provide a fair overview of how insurance coverage might be expected to vary across hospitals. We hypothesized that FP hospitals would admit a lower proportion of uninsured patients than NFP and government owned hospitals and a higher proportion of patients with private insurance and Medicare. We also expected that FP hospitals would be located in wealthier neighborhoods than NFP and government owned hospitals. Finally, we hypothesized that even after accounting for the socioeconomic conditions of the markets where hospitals of varying ownership were located, government hospitals would admit a significantly greater proportion of uninsured patients while FP hospitals would admit a significantly lower proportion of uninsured patients than NFP hospitals.

## Methods

### Data

We used state inpatient data (SID) for years 2005 from Arizona, Florida, Iowa, Massachusetts, Maryland, New Jersey, North Carolina, Washington, Wisconsin, and 2004 from New York to identify all patients age > 15 years hospitalized with acute myocardial infarction (AMI)(International Classification of Diseases, 9^th ^Clinical Modification [ICD-9-CM] code 410.× [N = 188,117]), coronary artery bypass grafting (CABG)(ICD-9 codes 36.10-36.19 [N = 82,261]), or childbirth (DRG codes 370-375 [N = 1,091,220]). The SID databases were developed by the Agency for Healthcare Research and Quality (AHRQ) as part of the healthcare utilization project (HCUP) in partnership with individual states (see: http://hcupnet.ahrq.gov/ for more information). SID data include many elements available on the UB-92 hospital discharge abstract, including demographics; primary and secondary diagnoses and procedures, as captured by ICD-9-CM codes; the diagnosis related group (DRG); admission source (categorized as emergency department; transfer from another hospital; transfer from sub-acute care; other); admission and discharge dates; patient's primary insurance as one of nine mutually exclusive categories; disposition at the time of hospital discharge (e.g., transfer to another acute care hospital; home; deceased); patient's zip code of residence; and a hospital specific identifier. We linked each hospital admitting patients with one of the three study conditions to the American Hospital Association (AHA) annual survey to obtain information on hospital governance (i.e., for-profit, not-for-profit or government ownership), bed size, and staffing levels. Hospitals (and associated patients) that could not be linked to the AHA data were excluded from the analysis (7 hospitals and 191 patients for AMI; 0 hospitals for CABG; 5 hospitals and 3,912 patients for Childbirth).

We linked each hospital address to zip code level data from the 2000 U.S. census to obtain socioeconomic measures for the neighborhoods (hospital service areas [HSAs]) surrounding each hospital; HSA's are defined geographic areas serviced by an individual hospital [29]. Specific socioeconomic measures used in the study included HSA-level: poverty rate defined as the proportion of people residing in each HSA with income below the federally specified threshold; family disruption as measured by the proportion of female heads-of-household who also had a child(ren) under 18 years of age; the proportion of male HSA residents between ages 16 to 64 years who were unemployed; and the proportion of people residing in the HSA who hold a managerial position. Each measure is standardized by the U.S. Census bureau to have a mean of 0 and standard deviation of 1.0 with more positive values connoting greater poverty, family disruption, and unemployment. We calculated hospital volume for each of the three study diagnoses by summing the number of patients admitted to each hospital using the SID data. We used AHA data to calculate nurse staffing ratios for each hospital as the number of nurse full-time equivalents (FTEs) divided by adjusted patient days [30].

### Analyses

We classified each hospital as FP, NFP, or government owned based upon the data obtained from the AHA survey. We used bivariate methods including the t-test and chi-square statistic to compare the demographic characteristics of patients admitted to FP, NFP, and government owned hospitals for each of the three patient cohorts. We used similar methods to compare differences in the urgency of each patient admission, the prevalence of selected comorbid conditions identified using the Quan modification of algorithms developed by Elixhauser et al,[31] and the percentage of childbirths that required cesarean section. We compared the characteristics of FP, NFP, and government owned hospitals including the mean annual number of admissions for each diagnosis, percentage of total hospitals classified as major teaching facilities, rural-urban location (as measured by rural-urban commuting area (RUCA)http://www.ers.usda.gov/Data/RuralUrbanCommutingAreaCodes, mean nurse staffing ratios, and the HSA level socioeconomic measures for the neighborhoods where FP, NFP, and government owned hospitals were located.

Next, we used similar bivariate methods to compare the insurance coverage of patients admitted to FP, NFP and government owned hospitals using the nine categories of insurance included in the SID data. Subsequent analyses combined the nine insurance groups into five categories based upon methods that we and others have used previously: Medicare; private insurance; Medicaid; uninsured (i.e., charity/self-pay); and other [32-34]. Our primary study endpoint was the percentage of patients admitted to FP, NFP, and government hospitals who were classified as uninsured. In order to allow us to compare the "amount" of uncompensated care provided by hospitals with varying ownership while accounting for differences in the relative wealth/poverty where hospitals were located, we developed patient-level generalized linear mixed models (GLMM) where the dependent variable was whether a particular patients was uninsured and the key independent variable of interest was hospital ownership (FP, NFP or government). For condition (CABG, AMI, or childbirth) we estimated three GLM models. The first (unadjusted) model included only patient insurance and hospital ownership. The second model adjusted only for hospital and market-level factors including the socioeconomic measures of each HSA, whether the hospital was located in an urban or rural area, and the state where each hospital was located. The third model adjusted for hospital and market-level factors plus patient-level factors including patient age (represented by indicator variables for ten-year increments), sex (included for AMI and CABG cohorts only), race (categorized as white, black, Hispanic, Asian/Pacific, Native American, and other), admission source (categorized as emergency department, inter-hospital transfer, nursing home, and other), and number of comorbid conditions that each patient had as identified using the algorithms of Quan et al. We then plotted the observed and adjusted distributions (i.e., mean, median, inter-quartile range) of the proportion of uninsured patients for NFP, FP, and government owned hospitals for each of the three patient cohorts.

The GLMM models assumed a binomial distribution and used a logit link function for uninsured admissions, and estimated fixed-effects of hospital ownership and covariates. In addition, the GLMM models incorporated random-effects for hospitals to adjust for the clustering of admissions within hospitals. All p-values are 2-tailed, with p-values less than .05 deemed statistically significant. All statistical analyses were performed using SAS 9.2 (SAS Institute Inc., Cary, NC). This project was approved by the University of Iowa Institutional Review Board.

## Results

Our study sample consisted of 188,117 patients admitted for AMI to 1,054 hospitals, 82,261 patients admitted for CABG to 245 hospitals, and 1,091,220 patients admitted for childbirth to 793 hospitals. Patients admitted to FP, NFP, and government hospitals differed in a number of ways (Table 1). For example, patients admitted to FP hospitals for AMI and CABG tended to be older than patients admitted to NFP and government owned hospitals, while patients admitted to FP hospitals for childbirth tended to be younger (P < .001 for all comparisons). Likewise, patients admitted to FP hospitals for AMI and CABG were more likely to be white than patients admitted to NFP and government hospitals though this was not the case for childbirth. Government hospitals admitted the highest proportion of black patients for each of the study diagnoses while for-profit hospitals admitted the highest proportion of Hispanic patients for AMI and CABG but not childbirth. FP hospitals admitted a higher proportion of patients through the emergency department for both AMI and CABG and had slightly higher c-section rates for childbirth (P < .001). Patients admitted to FP hospitals for AMI and CABG tended to have higher rates of most comorbid conditions (P < .05) though this was not the case for childbirth.

**Table 1:** Characteristics of patients hospitalized in for-profit, not-for-profit, and government owned hospitals for AMI, CABG and Childbirth

		AMI (N = 188117)			CABG (N = 82261)			Childbirth (N = 1091220)		
		**NFP** **N = 146309**	**FP** **N = 23134**	**Government** **N = 18674**	**NFP** **N = 66513**	**FP** **N = 8947**	**Government** **N = 6801**	**NFP** **N = 864954**	**FP** **N = 95406**	**Government** **N = 130860**

Age										

Years, mean (sd)		69.1^∧^ (14.7)	70.0^∧^ (14.6)	67.4^∧^ (15.0)	67.1^∧^ (10.8)	67.7^∧^ (10.7)	65.9^∧^ (11.0)	28.3^∧^ (6.2)	26.8^∧^ (6.1)	27.4^∧^ (6.2)


Gender										

Female, number (%)		61919^‡^ (42.3)	9544* (41.3)	7641^∧^ (40.9)	19098* (28.7)	2553* (28.5)	1877* (27.6)	NA	NA	NA


Race										

White, number (%)		103169^∧^ (70.5)	16709^∧^ (71.9)	10482^∧^ (56.1)	47141^∧^ (70.9)	7158^∧^ (80.0)	4936^‡^ (72.6)	426164^∧^ (49.3)	44059^∧^ (45.7)	37247^∧^ (28.4)

Black, number (%)		10460^∧^ (7.2)	1805^∧^ (7.8)	2296^∧^ (12.3)	2901^∧^ (4.4)	309^∧^ (3.5)	548^∧^ (8.1)	105164^∧^ (12.1)	15469^∧^ (16.0)	23303^∧^ (17.8)

Hispanic, number (%)		7326^∧^ (5.0)	3104^∧^ (13.4)	850^‡^ (4.6)	2948^∧^ (4.4)	781^∧^ (8.7)	342^†^ (5.0)	127197^∧^ (14.7)	26006^∧^ (27.0)	16271^∧^ (12.4)

Asian/Pacific, number (%)		1419^∧^ (1.0)	112^∧^ (0.5)	187* (1.0)	642^∧^ (1.0)	50* (0.6)	48^†^ (0.7)	31528^∧^ (3.6)	1682^∧^ (1.7)	3299^∧^ (2.5)

Native American, number (%)		512^‡^ (0.4)	56* (0.2)	46^†^ (0.3)	275^†^ (0.4)	22* (0.3)	19* (0.3)	6763^∧^ (0.8)	360^∧^ (0.4)	617^∧^ (0.5)

Other, number (%)		23423^∧^ (15.9)	1348^∧^ (5.8)	4813^∧^ (25.8)	12606^∧^ (18.9)	627^∧^ (7.0)	908^∧^ (13.4)	168138^∧^ (19.4)	8864^∧^ (9.2)	50123^∧^ (38.3)


Admission Source										

Emergency Department, number (%)		96067^∧^ (65.78)	17366^∧^ (75.1)	12339* (66.1)	13548^∧^ (20.4)	2151^∧^ (24.0)	1453* (21.4)	41734^∧^ (4.8)	3091^∧^ (3.2)	23711^∧^ (18.1)

Inter-hospital transfer, number (%)		26255^∧^ (17.9)	2388^∧^ (10.3)	2491^∧^ (13.3)	12480^∧^ (18.8)	1284^∧^ (14.4)	1182^‡^ (17.4)	3118^∧^ (0.4)	123^∧^ (0.1)	567^∧^ (0.4)

Nursing Home, number (%)		4790* (3.3)	704^∧^ (3.0)	310^∧^ (1.7)	1497^∧^ (2.3)	297^∧^ (3.3)	129* (1.9)	3187^∧^ (0.4)	91* (0.1)	115^∧^ (0.1)

Other, number (%)		19197^∧^ (13.1)	2676^∧^ (11.5)	3534^∧^ (18.9)	38988* (58.6)	5215* (58.3)	4037* (59.4)	816915^∧^ (94.4)	90101^∧^ (96.6)	106467^∧^ (81.4)


Type of Delivery										

Vaginal, number (%)		NA	NA	NA	NA	NA	NA	596163^∧^ (68.9)	64721^∧^ (67.8)	90576^†^ (69.2)

C-Section, number (%)		NA	NA	NA	NA	NA	NA	268791^∧^ (31.1)	30685^∧^ (32.2)	40284^†^ (30.8)


Comorbidity										

COPD, number (%)		30477^∧^ (20.8)	5536^∧^ (24.0)	3746^†^ (20.1)	14417^∧^ (21.7)	2236^∧^ (25.0)	1352^∧^ (19.9)	23182^∧^ (2.7)	1532^∧^ (1.6)	3223^∧^ (2.5)

Diabetes Uncomplicated, number (%)		37207^†^ (25.4)	5728^∧^ (24.8)	5017^∧^ (26.9)	18284* (27.5)	2379^‡^ (26.6)	1950^†^ (28.7)	5243^∧^ (0.6)	454^∧^ (0.5)	969^∧^ (0.7)

Renal failure, number (%)		13948^∧^ (9.5)	2652^∧^ (11.4)	1692^†^ (9.0)	4160* (6.3)	607* (6.8)	474^†^ (7.0)	132* (0.0)	9^†^ (0.0)	31^†^ (0.0)

Obesity, number (%)		9614* (6.6)	1478* (6.4)	1110^‡^ (5.9)	5787* (8.7)	726* (8.1)	524* (7.7)	9485^∧^ (1.1)	627^∧^ (0.7)	1842^∧^ (1.4)

Depression, number (%)		6983^∧^ (4.8)	920* (4.0)	812^‡^ (4.4)	2195^∧^ (3.3)	223^∧^ (2.5)	240* (3.5)	13089^∧^ (1.5)	873^∧^ (0.9)	1945* (1.5)

*: >0.05

†: ≤0.05

‡: <0.01

∧: <0.001

Symbol in NFP cell represents P-value for NFP vs. FP

Symbol in FP cell represents P-value for FP vs. Government

Symbol in Government cell represents P-value for Government vs. NFP

FP, NFP, and government owned hospitals differed with regard to many structural and organizational characteristics (Table 2). NFP hospitals were, on average, larger than both FP and government owned hospitals as measured by both admission volume and number of licensed beds though not all comparisons reached statistical significance. FP hospitals were less likely to be major teaching hospitals and more likely to be located in urban areas when compared to NFP and government hospitals. FP hospitals also had a significantly higher nurse staffing ratio than NFP and government hospitals. There was little consistent difference in socioeconomic measures of the neighborhoods where NFP, FP, and government hospitals were located.

**Table 2:** Structural characteristics of for-profit, not-for-profit, and government owned hospitals and neighborhoods where these hospitals are located

	AMI (N = 1054)			CABG (N = 245)			Childbirth (N = 793)		
	
	NFP	FP	Government	NFP	FP	Government	NFP	FP	Government
Number of hospitals	732	140	182	185	39	21	586	73	134

Number of Admissions, mean (sd)	219* (288)	179* (204)	111^∧^ (215)	359* (261)	229^†^ (117)	324* (182)	1476^‡^ (1597)	1307* (953)	977* (1292)

Hospital Beds, mean (sd)	220^†^ (224)	163* (108)	173^†^ (276)	433^∧^ (275)	257^∧^ (115)	641^‡^ (302)	246* (237)	187* (103)	183^†^ (241)

Major teaching hospitals, number (%)	77^∧^ (10.4)	0^∧^ (0.0)	25* (13.7)	59^∧^ (30.7)	0^∧^ (0.0)	11^†^ (52.4)	70^‡^ (12.0)	0^∧^ (0.0)	23* (17.2)


Rural/Urban Location									

Rural, number (%)	58 (7.9)	3^∧^ (2.1)	29 (15.9)	1 (0.5)	0 (0.0)	0 (0.0)	35 (6.0)	1^†^ (1.4)	14* (10.5)

Suburban, number (%)	230^‡^ (31.4)	25^∧^ (17.9)	92^∧^ (50.6)	13 (6.8)	0 (0.0)	1 (4.8)	191^†^ (32.6)	15^∧^ (20.6)	73^∧^ (54.5)

Urban, number (%)	444^∧^ (60.7)	112^∧^ (80.0)	61^∧^ (33.5)	178 (92.7)	39 (100.0)	20 (95.2)	360^‡^ (61.4)	57^∧^ (78.1)	47^∧^ (35.1)


Nurse Staffing Ratio, mean (sd)	2.47^∧^ (1.07)	3.14^∧^ (1.31)	2.56* (1.7)	3.12* (0.95)	3.51* (1.82)	3.16* (0.92)	2.54^†^ (1.06)	3.22^∧^ (0.95)	2.78* (1.82)


Socioeconomic Measures									

Poverty (standardized), mean (sd)	-0.26^†^ (0.79)	-0.04* (0.72)	-0.4^‡^ (0.89)	-0.17* (0.76)	-0.04* (0.68)	0.08* (0.86)	-0.29^∧^ (0.84)	-0.05* (0.76)	0.01^†^ (0.94)

Family Disruption, (standardized), mean (sd)	0.15* (1.08)	0.29* (0.85)	0.07* (1.21)	0.41* (1.03)	0.35* (0.82)	0.44* (1.26)	0.17* (1.08)	0.33* (0.85)	0.10* (1.25)

Unemployment Rate (standard-ized), mean (sd)	-0.11^‡^ (0.88)	0.17* (0.74)	-0.03* (1.04)	-0.07* (0.78)	0.06* (0.67)	0.18* (0.82)	-0.15* (0.88)	0.13* (0.74)	0.01* (1.07)

Occupational Composition (standardized), mean (sd)	-0.46^∧^ (1.07)	0.02* (0.94)	-0.20^‡^ (0.98)	-0.76^∧^ (0.98)	-0.06^†^ (0.77)	-0.70* (1.26)	-0.47^∧^ (0.98)	0.09* (0.86)	-0.20^†^ (0.98)

*: >0.05

†: ≤0.05

‡: <0.01

∧: <0.001

Symbol in NFP cell represents P-value for NFP vs. FP

Symbol in FP cell represents P-value for FP vs. Government

Symbol in Government cell represents P-value for Government vs. NFP

In our analysis of insurance coverage, a significantly higher proportion of patients admitted to FP hospitals were insured by Medicare as compared with NFP and government owned hospitals for each of the study diagnoses (Table 3); this difference was particularly striking for CABG (93.4% Medicare for FP; 57.9% for NFP; and 54.3% for government; P < .001). Alternatively, patients admitted to NFP were more likely to be privately insured and patients admitted to government hospitals were more likely to be insured by Medicaid for each of the three study diagnoses (P < .001). Patients admitted to NFP hospitals were significantly less likely to be classified as uninsured (i.e., either charity care or self-pay) than patients admitted to either FP or government owned hospitals for each of the three study conditions (P < .001); for example, 4.6% of all patients admitted to NFP hospitals with AMI were classified as uninsured as compared to 6.0% of patients admitted to FP hospitals and 9.5% of patients admitted to government owned hospitals (P < .001).

**Table 3:** Insurance coverage of patients treated in for-profit, not-for-profit, and government owned hospitals

	AMI (N = 188117)			CABG (N = 82261)			Childbirth (N = 1091220)		
	
	NFP N = 146309	FP N = 23134	Government N = 18674	NFP N = 66513	FP N = 8947	Government N = 6801	NFP N = 864954	FP N = 95406	Government N = 130860
Insurance Type									

Medicare FFS, number (%)	57679 (39.4)	11039 (47.5)	5686 (30.5)	24475 (36.8)	4120 (46.1)	2436 (35.8)	1979 (0.2)	297 (0.3)	226 (0.2)

Medicare FFS/MC, number (%)	22968 (15.6)	698 (3.4)	3801 (2.4)	9628 (14.4)	330 (3.7)	855 (12.6)	613 (0.1)	27 (0.0)	196 (0.2)

Medicare MC, number (%)	8761 (6.0)	3269 (14.1)	1115 (6.0)	4390 (6.6)	1221 (13.7)	405 (6.0)	1185 (0.1)	31 (0.0)	17 (0.0)

Medicare Total	89404^∧^ (61.1)	15006^∧^ (64.9)	10602^∧^ (56.8)	38493^∧^ (57.9)	5671^∧^ (93.4)	3696^∧^ (54.3)	3805^‡^ (0.4)	355* (0.4)	439^∧^ (0.3)


Private FFS/PPO, number (%)	23266 (15.9)	2719 (11.8)	3080 (16.5)	12477 (18.8)	1240 (13.9)	1249 (18.4)	264233 (30.6)	16579 (17.4)	32661 (25.0)

Private HMO, number (%)	17213 (11.8)	2349 (10.1)	1213 (6.5)	9690 (14.5)	1107 (12.4)	706 (10.4)	244412 (28.3)	21636 (22.7)	13325 (10.2)

Private Total	40479^∧^ (27.7)	5068^‡^ (21.9)	4293^∧^ (23.0)	22167^∧^ (33.3)	2347^∧^ (26.2)	1955^∧^ (28.8)	513512^∧^ (58.9)	38902^∧^ (40.3)	45986^∧^ (35.1)


Medicaid, number (%)	7489^†^ (5.1)	1112^∧^ (4.8)	1627^∧^ (8.7)	3010^†^ (4.5)	357^∧^ (4.0)	496^∧^ (7.3)	310577^∧^ (35.9)	49830^∧^ (52.2)	66641^∧^ (50.1)


Self Pay, number (%)	5794 (4.0)	1013 (4.4)	1473 (7.9)	1462 (2.2)	231 (2.6)	312 (4.6)	25278 (2.9)	3649 (3.8)	14272 (10.9)

Charity, number (%)	857 (0.6)	379 (1.6)	301 (1.6)	266 (0.4)	68 (0.8)	165 (2.4)	1760 (0.2)	370 (0.4)	1126 (0.9)

Uninsured Total	6651^∧^ (4.6)	1392^∧^ (6.0)	1774^∧^ (9.5)	1728^∧^ (2.6)	299^∧^ (3.3)	477^∧^ (7.0)	27038^∧^ (3.1)	4019^∧^ (4.2)	15398^∧^ (11.8)


Other, number (%)	2282^∧^ (1.6)	556^†^ (2.4)	378^∧^ (2.0)	1115^∧^ (1.7)	273* (3.1)	177^∧^ (2.6)	14917^∧^ (1.7)	2988^∧^ (3.1)	2396^‡^ (1.8)

*: >0.05

†: ≤0.05

‡: <0.01

∧: <0.001

Symbol in NFP cell represents P-value for NFP vs. FP

Symbol in FP cell represents P-value for FP vs. Government

Symbol in Government cell represents P-value for Government vs. NFP

**Figure 1:**
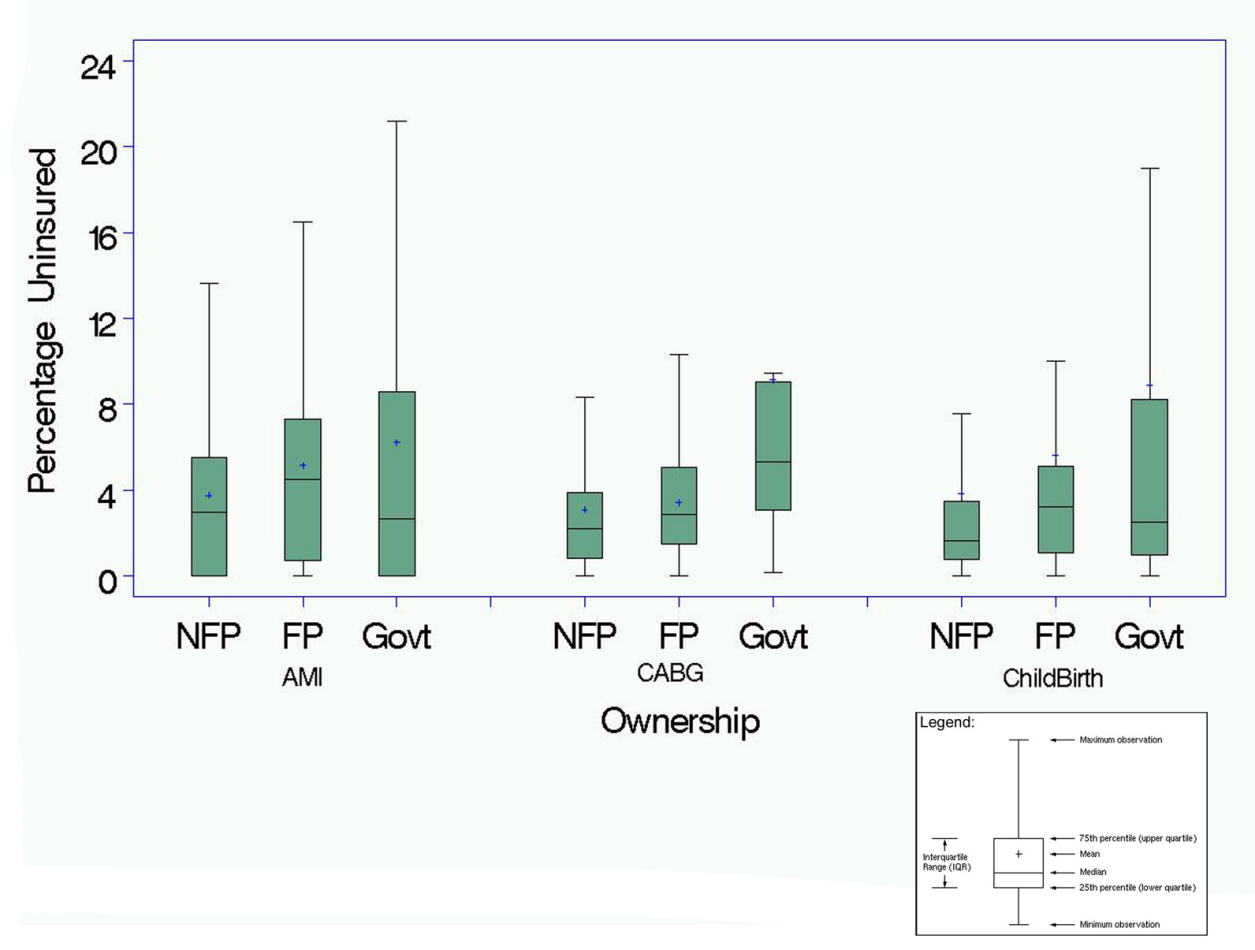
**The observed (unadjusted) percentage of uninsured patients for all not-for-profit (NFP), for-profit (FP) and government owned hospitals**.

**Figure 2:**
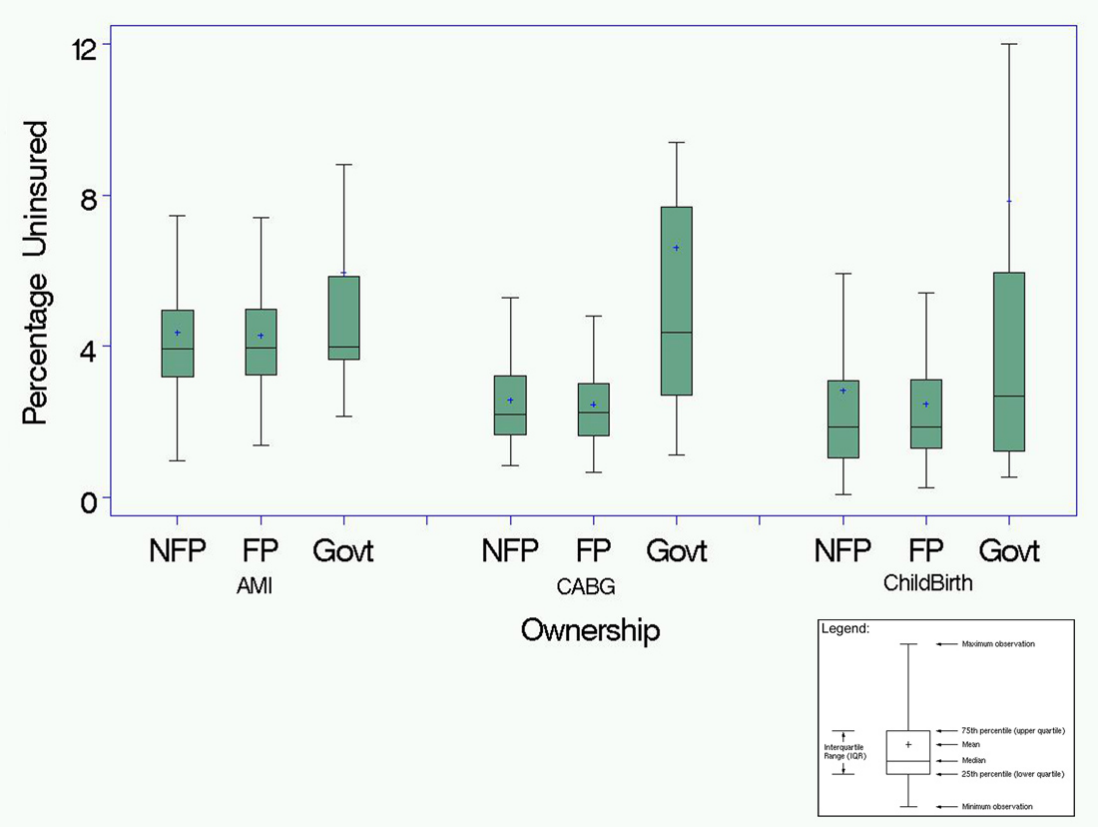
The percentage of uninsured patients for all not-for-profit (NFP), for-profit (FP) and government owned hospitals after adjusting for market level socioeconomic factors and clustering of patients within hospitals*.

**Figure 3:**
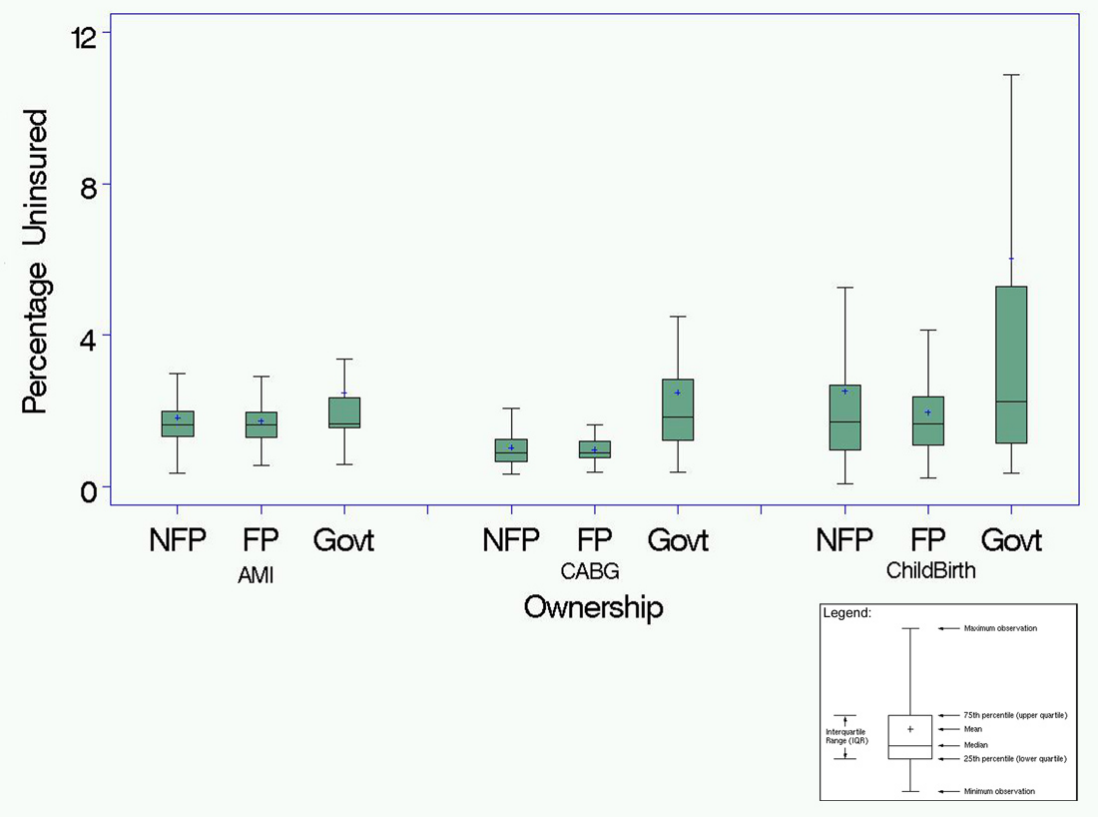
The percentage of uninsured patients for all not-for-profit (NFP), for-profit (FP) and government owned hospitals after adjusting for market level socioeconomic factors, patient demographics and comorbidity, and clustering of patients within hospitals*.

In unadjusted analyses (Figure 1), the mean percentage of uninsured patients hospitalized with AMI was 3.8% for NFP hospitals, 5.2% for FP hospitals (P < .05 for NFP vs. FP), and 6.2% for government-owned hospitals (P < .01 for NFP vs. government); for CABG the percentage of uninsured was 3.1% for NFP hospitals, 3.4% for FP hospitals (P = 0.56 for NFP vs. FP), and 9.2% for government-owned hospitals (P < .05 for NFP vs. government); and for childbirth the percentage of uninsured was 3.8% for NFP hospitals, 5.6% for FP hospitals (P = 0.23 for NFP vs. FP hospitals), and 8.9% for government-owned hospitals (P < .001 for NFP vs. government). In regression models that adjusted for hospital and market-level characteristics (Figure 2), the mean percentage of uninsured patients hospitalized with AMI was 4.4% for NFP hospitals, 4.3% for FP hospitals (P = 0.71 for NFP vs. FP), and 6.0% for government-owned hospitals (P < .001 for NFP vs. government); for CABG the percentage of uninsured was 2.6% for NFP hospitals, 2.5% for FP hospitals (P = 0.57 for NFP vs. FP), and 6.6% for government-owned hospitals (P <.01 for NFP vs. government); and for childbirth the percentage of uninsured was 2.8% for NFP hospitals, 2.5% for FP hospitals (P = 0.27 for NFP vs. FP hospitals), and 7.9% for government-owned hospitals (P < .001 for NFP vs. government). In additional models that adjusted for patient characteristics as well (Figure 3), we again found that government hospitals admitted a higher proportion of uninsured patients while FP and NFP hospitals admitted similar levels of uninsured for all three diagnoses.

## Discussion and Conclusions

In an analysis of state all-payor data we found that, on-average, not for-profit hospitals provide a similar level of uncompensated care as for-profit hospitals for patients hospitalized with three common diagnoses. These findings are noteworthy given the growing concern that certain NFP hospitals may not provide enough charity care to justify the generous tax exemptions that they receive. Alternatively, our finding that government hospitals provide significantly more uncompensated care than either NFP or FP hospitals is a testimony to the vital role that these hospitals play the U.S. healthcare system.

A number of aspects of our study merit further discussion. First, it is important to explain why the tax exempt status of not-for-profit hospitals is so important. America's approximately 4,200 NFP hospitals receive an estimated $6-$8 billion in tax exemptions per year (1995 dollars)-- an average of $1.6 million per hospital [35]. It is presumed by policy makers that these tax savings will be used by NFP hospitals to support activities that provide so-called "community benefit" [36,37]. Community benefit, while difficult to quantify, typically encompasses a range of activities including provision of care to the uninsured, community outreach activities, research, and teaching [36,18,38]. Quantifying community benefit is methodologically challenging because many components (e.g., hospital outreach activities, research, and teaching) are not readily and reliably captured in commonly available data sources. Thus, in practice, most research and policy evaluations of the quantity of community benefit provided by hospitals typically relies upon study of the amount of charity care provided to the underinsured and uninsured. There are a relatively limited number of data sources that contain data on the quantity of charity care provided by hospitals; these sources include surveys collected by hospital organizations (e.g., the AHA annual survey) and financial reports derived from information that hospitals are required to submit to state and federal regulators (e.g., Internal Revenue Service Schedule H for hospitals)[2,26,27,39]. While these data are certainly useful, there is emerging evidence that hospitals are not so different from private sector businesses when it comes to the pressures to manipulate financial data in unseemly ways [28]. In particular, NFP hospitals have recognized that their financial statements are scrutinized by regulators and the media. Hospitals with excessive profitability or minimal amounts of charity care in these reports are increasingly targeted for financial audits and even penalties. Given these incentives to "manage" earnings, we believe we believe that our analysis-focusing on the actual insurance coverage of patients admitted to hospitals as opposed to hospital financial statements-provides an important new method for evaluating charity care provided by hospitals.

Second, it is important to discuss our finding that NFP hospitals do not appear to provide a greater level of uncompensated care than FP hospitals. We initially suspected that the lower proportion of uninsured patients admitted to NFP hospitals in unadjusted analyses might reflect the neighborhoods where not-for-profit hospitals were located [26]. However, even after adjusting for patient, hospital, and market-level factors we found no evidence that NFP hospitals provide a greater level of uncompensated care than FP hospitals. Thus, other explanations must be explored. One potential explanation is that uninsured patients preferentially seek care from for-profit and government owned hospitals [40,41]. It is easy to understand why uninsured patients might prefer government operated safety net hospitals that often have an explicit mission to provide medical care to vulnerable populations, but it is less clear why uninsured patients would prefer for-profit hospitals. Another possible explanation for our finding that not-for-profit hospitals perform less charity care comes from the work of Weiner et al. who have found evidence that hospitals have implicit policies and strategies to minimize the amount of care provided to uninsured populations [42].

Third, it is important to consider our findings in the context of how we defined uninsured patients in this analysis. We defined uninsured patients as those patients whose payment source was categorized as either charity care or self-pay in the SID data in accordance with methods that have been employed in prior studies [43]. There is some evidence that these two groups of patients differ with individuals classified as self-pay being more likely to represent either individuals who have declined employer sponsored health insurance or the so-called working poor while individuals categorized as charity care may be more likely to represent the truly indigent [44,45]. That said, from a hospital perspective both charity care and self-pay patients constitute populations for whom hospitals are less likely to receive adequate reimbursement for services that are provided and thus combining these groups for analytic purposes seems justified [46,47].

Finally, we would like to briefly comment on two additional findings. Our finding that for-profit hospitals had higher nurse staffing levels than both not-for-profit and government owned hospitals is important. While conventional wisdom and older studies have suggested that that for-profit hospitals may reduce nurse staffing as a strategy to maximize profits more recent studies have demonstrated a more complex picture with reduced levels of nurse staffing in both for-profit and not-for-profit hospitals. Our finding of higher nurse staffing levels in for-profit hospitals in combination with our principal findings that for-profit hospitals do not admit fewer uninsured patients adds to evidence that for-profit hospitals may not place profitability above other important aspects of hospital quality. It is also important to briefly comment on our finding that more than 40% of all women admitted for childbirth were primarily insured by Medicaid. While on first glance this appears extremely high, these data are consistent with prior studies and likely reflect a combination of the erosion of employer based health insurance combined with the socioeconomic challenges faced by younger mothers.

There are a number of limitations to our study that merit brief mention. First, our study relied upon administrative data and thus may have been subject to bias if insurance status was systematically miscoded more often by one group of hospitals, though we have no reason to suspect this to be the case. Second, our analysis was limited to patients admitted with three different diagnoses to hospitals in ten states and therefore should be extrapolated to other diagnoses and states with caution. Further study is needed to verify the generalizability of our study to other conditions. Third, our study focused on amount of community benefit provided by hospitals as measured by the proportion of admissions who were categorized as uninsured and did not assess other aspects of community benefit that are important to consider. Nevertheless, in practice community benefit is commonly assessed through the measurement of charity care or uncompensated care and thus we believe that our analysis is germane to the current political debate.

In summary, we found no evidence that not-for-profit hospitals admit a higher percentage of uninsured patients than for-profit or government-owned hospitals, for the three conditions studied. While we cannot comment on other types of community benefits that not-for-profit hospitals may provide, concerns about the tax exempt status of not-for-profit hospitals may be warranted.

## Competing interests

Dr. Vaughan-Sarrazin is a Research Scientist in the Center for Research in the Implementation of Innovative Strategies in Practice (CRIISP) at the Iowa City VA Medical Center, which is funded through the Department of Veterans Affairs, Veterans Health Administration, Health Services Research and Development Service. Dr. Cram is supported by a K23 career development award (RR01997201) from the NCRR at the NIH and the Robert Wood Johnson Physician Faculty Scholars Program. This work is also funded by R01 HL085347-01A1 from NHLBI at the NIH. The views expressed in this article are those of the authors and do not necessarily represent the views of the Department of Veterans Affairs. The funding sources had no role in the analyses or drafting of this manuscript. In 2010 Dr. Cram was paid $2,500 for advising Vanguard Health Inc. on quality improvement efforts. None of the authors have any conflicts of interest.

## Authors' contributions

LB conducted all study analyses. PC, MSVS, and XC oversaw and supervised the analyses. PC, MSVS, and GER developed the idea for the study, interpreted the results, drafted and revised the manuscript. All authors have read and approved this paper.

## Pre-publication history

The pre-publication history for this paper can be accessed here:

http://www.biomedcentral.com/1472-6963/10/90/prepub
